# Expression Pattern of Myelin-Related Apolipoprotein D in Human Multiple Sclerosis Lesions

**DOI:** 10.3389/fnagi.2018.00254

**Published:** 2018-08-21

**Authors:** Ana Navarro, Beatriz Rioseras, Eva del Valle, Eva Martínez-Pinilla, Aurora Astudillo, Jorge Tolivia

**Affiliations:** ^1^Instituto de Neurociencias del Principado de Asturias (INEUROPA), Asturias, Spain; ^2^Instituto de Investigación Sanitaria del Principado de Asturias (ISPA), Asturias, Spain; ^3^Departamento de Morfología y Biología Celular, Facultad de Biología y Medicina, Universidad de Oviedo, Oviedo, Spain; ^4^Servicio de Anatomía Patológica, Hospital Universitario Central de Asturias, Oviedo, Spain

**Keywords:** demyelination, remyelination, myelin, axon loss, oligodendrocytes, neurodegenerative diseases

## Abstract

Apolipoprotein D (Apo D) is a key molecule in the lipid transport during homeostasis and repair processes in normal and pathological conditions of the nervous system with a putative neuroprotective effect. In the last decades, huge experimental efforts have been made to know the exact mechanism of action of Apo D, even though, it remains an open question. In this regard, studies in mammals and flies have suggested that Apo D seems to act through a variety of cellular mechanisms related with its ability to selectively bind different lipid ligands. For instance, this apolipoprotein is required to myelin compaction, it participates in axon regeneration/remyelination, and it can control the magnitude and timing of the inflammatory response after injury, promoting myelin clearance, and regulating the number of immune cells recruited to the damaged area. These, among others, are some of the reasons to study Apo D in multiple sclerosis (MS) pathology, where it could be particularly important since the autoimmune reaction against oligodendrocytes (OLGs) and myelin is generally assumed as the most plausible cause of this pathology. The aim of this work was to investigate the Apo D expression pattern in MS lesions, including active and inactive demyelinating plaques, and also remyelinating ones. Human brain tissues with inflammatory demyelination consistent with MS were used to quantify Apo D immunosignal in different lesions. Our results show a clear decrease of Apo D expression in all sclerosis plaques, being lower in the inactive than in active areas but recovers in the remyelination ones. Apo D is mainly produced by the matured OLGs of white matter and is located in cell processes surrounding the myelin sheath. All these data seem to indicate an important role of Apo D in myelination/remyelination processes as a molecule with a neuroprotective potential, and may serve as a good starting point for its study in MS.

## Introduction

Multiple sclerosis (MS) is a chronic, neurodegenerative, and demyelinating disease of the central nervous system (CNS) that affects to more than 2.1 million people worldwide and for which there is still no cure (Ramagopalan et al., 2010). The main processes that characterize MS are demyelination and axonal damage followed, in some cases, by remyelination events in an attempt to restore the induced damage. The exact cause of MS remains unknown, but an exacerbated autoimmune reaction against oligodendrocytes (OLGs) and myelin protein, and the resulting chronic inflammation have been postulated as the most plausible origins (McQualter and Bernard, 2007; Zindler and Zipp, 2010).

In MS, autoreactivity to different myelin proteins involves cellular and humoral immunity. It has been described that a defective and permeable blood-brain barrier (BBB) can lead to some T lymphocytes to infiltrate the brain parenchyma, secrete proinflammatory cytokines, and stimulate monocytes/macrophages that promote damage of myelin sheaths and the loss of OLGs (McQualter and Bernard, 2007; McGuire et al., 2011). For its part, the humoral response implies the activation of B lymphocytes with the secretion of anti-myelin antibodies (Disanto et al., 2012). The main consequence of the inflammation-mediated demyelination is an acute axonal degeneration. Thus, limitation of electrical impulses can be manifested as a decrease in conduction speed, a failure to transmit the action potential at high frequencies, or as a total conduction blockage leading to neurological dysfunction (Fuller and Goodman, 2001).

MS focal demyelinating lesions, known as plaques, appear as scars in the white matter (WM) throughout the brain and spinal cord (Zindler and Zipp, 2010). Often, these pathological features evolve differently during disease phases and, even, plaques in different stages of demyelinating activity are evident in the same phase. The active plaques characterized by the presence of activated mononuclear cells, OLGs, and myelin debris are common in the acute phase. For its part, chronic inactive and completely demyelinated plaques appear as the MS progresses; they show minor cellular infiltration and a substantial loss of axons and OLGs (Popescu et al., 2013). Under specific circumstances and in some sclerosis plaques, remyelination can occur (shadow plaques). The entire myelin sheath is restored in demyelinated axons, reinstating saltatory conduction, and resolving functional deficits. This transient process is largely due to the so-called OLGs precursor cells (OPCs) that are recruited to the damaged areas (Frohman et al., 2006).

Apolipoprotein D (Apo D) belongs to the lipocalin family whose members share the ability to bind and transport small hydrophobic ligands, that is, arachidonic acid, cholesterol, or steroids, due to their conserved tertiary structure with a ligand-binding pocket (Rassart et al., 2000; Ruiz et al., 2013). This 29 kDa glycoprotein is ubiquitously expressed in neural and peripheral tissues, detected in cerebrospinal fluid (CSF) and as an important component of high-density plasma lipoproteins (HDL) (Rassart et al., 2000). In the peripheral nervous system (PNS), Schwann cells contribute to local synthesis of Apo D and its expression is strongly induced after nerve injury (Spreyer et al., 1990; Ganfornina et al., 2010; García-Mateo et al., 2014, 2018). Furthermore, axonal regeneration and remyelination are delayed in Apo D knock out (KO) mice, which proves, unequivocally, the apolipoprotein involvement in myelination processes (Ganfornina et al., 2010).

In non-pathological conditions of the CNS, Apo D expression is always greater in the WM than in the gray one and is widely expressed in many nervous cells such as periventricular cells, neurons, OLGs, and astrocytes (Navarro et al., 1998, 2010; Hu et al., 2001). Noteworthy, Apo D is progressively accumulated in neurons and astrocytes during aging, in several types of CNS injury and neurodegenerative diseases as Parkinson, spongiform encephalopathy, Niemann-Pick, or Alzheimer’s disease (AD) (Muffat and Walker, 2010; Dassati et al., 2014). Evidence supports a correlation between this Apo D up-regulation and lipid transport during homeostasis and repair processes in normal and pathological conditions. In this sense, it expression in the cytoplasm and in OLGs processes of the WM seems to be crucial for the myelin sheath formation during development (Navarro et al., 2004). In recent decades, there is evidence of the potential of Apo D as neuroprotective molecule, preventing lipid peroxidation and excitotoxic injury (Ganfornina et al., 2008; Bajo-Grañeras et al., 2011), and contributing to neuronal trophism (Kosacka et al., 2009). Interestingly, those neurons most vulnerable to death are unable to synthesize or capture Apo D (Ordoñez et al., 2006).

Data from proteomic studies have currently suggested that various apolipoproteins, Apo D and Apo E among them, are involved in MS; elevated levels of these proteins were found in the CSF of MS patients (Stoop et al., 2010). Unfortunately, there are few studies on evaluating the implication of Apo D in the pathology, and in some cases, the results are seemingly contradictory (Reindl et al., 2001; Kroksveen et al., 2012). The aim of the present study was to analyze, for first time to or knowledge, the Apo D expression pattern in focal areas of myelin loss and its relationship with demyelination and remyelination processes in MS, in an attempt to know the exact role of Apo D in the pathology.

## Materials and Methods

### Subjects

The present study was conducted according to the Declaration of Helsinki, and it has been approved by the local Research Ethics Committee of Principado de Asturias.

Human brain and spinal cord tissues were provided by several Spanish brain banks: Biobank of the University Central Hospital of Asturias (*n* = 3), Barcelona University-Clinical Hospital (*n* = 6), Central Bank Madrid (CIEN Foundation) (*n* = 1), and Navarra Hospital (*n* = 4). Eleven subjects between 27 and 68 years old with histologically proven inflammatory demyelination consistent with MS, properly confirmed by a neuropathologist, were used in this study (**Table 1**). The ethics committees of each participating biobank have reviewed and approved the study protocol. Whereas most biobanks waived the need for informed consent due to the anonymous and non-interventional fashion of this study, a formal written consent obtained from patients or surrogates was an essential requirement for other ones.

**Table 1:** Demographic data of patients.

Case	Age	Sex	Cause of death	Time postmortem	Type of MS	Number of samples	Brain area	Type of lesion
1	58	Male	Cardiogenic shock	<12 h	PPMS	1	Periventricular WM	A big chronic-active plaque and little active plaques
2	65	Female	Cardiorespiratory arrest	<12 h	SPMS	1	Periventricular WM	Chronic-active plaques with remyelination
3	48	Female	Cardiorespiratory arrest	<12 h	Long-term SPMS	1	Subcortical WM of the temporal lobe	Chronic-active plaques
4	46	Male	Multiorgan failure	<12 h	PPMS	1	Periventricular WM	Chronic-active plaques
5	68	Male	Multiorgan failure	<12 h	SPMS	1	Periventricular WM	Chronic-active plaques with remyelination
6	52	Female	Cardiorespiratory arrest	<12 h	SPMS	1	Periventricular WM	Chronic-active plaques
7	64	Male	Acute myocardial infarction	<12 h	Long-term SPMS	1	Periventricular WM	Chronic-active plaques with remyelination
8	?	?		<12 h	Long-term SPMS	1	Subcortical WM of the temporal lobe	Chronic-active plaques
9	27	Female	Multiorgan failure	6–12 h	Long-term SPMS	3	Subcortical WM of the frontal lobe, brain stem, spinal cord	Little active plaques
10	38	Male	Respiratory failure	6–12 h	SPMS	3	Subcortical WM of the frontal and parietal lobes, spinal cord	Chronic-active plaques
11	36	Female	Multiorgan failure	6–12 h	Long-term SPMS	3	Subcortical WM of the frontal, parietal and temporal lobes	Chronic inactive plaques
12	79	Male	Multiorgan failure	6–12 h	Control	1	Spinal cord	
13	66	Male	Cardiorespiratory arrest	<12 h	Control	1	Periventricular WM	
14	65	Male	Cardiorespiratory arrest	<12 h	Control	1	Subcortical WM of the occipital lobe	

*MS, multiple sclerosis; PPMS, primary progressive MS; SPMS, secondary progressive MS; WM, white matter*.

Human brain and spinal cord samples were fixed by immersion in 10% buffered formalin. After fixation, pieces were dehydrated, cleared in butyl acetate, and embedded in paraffin. Sections about 5–7 μm thick were obtained, mounted on “SuperFrost Plus” (Mentzel-Glasse) slides, and dried at 36°C for 24 h.

### Cytoarchitectonic Staining

To visualize the morphological features and the different cell types in the studied regions, sections were desparaffined in xylene, and partially hydrated by successive alcohols. Then, and in order to locate demyelination plaques and determine lesion activity, sections were stained using hematoxylin-eosin, luxol fast blue (LFB) myelin stain, periodic acid–Schiff (PAS), and ferric hematoxylin, a technique developed in our laboratory (Tolivia et al., 1988), which specifically stains myelin without differentiation process. This technique is a simple method for the differential staining of myelinated nerve fibers (blue/black color) and cell bodies (red color) in two steps; first step with ferric hematoxylin and second with pyronine (Tolivia et al., 1988). According demyelinating activity (Lucchinetti et al., 1999; Bramow et al., 2010; Popescu et al., 2013), the following stages were defined: active areas located at the plaque border between demyelinated plaque and periplaque WM, inactive areas that showed a complete demyelination and, finally, remyelinating areas characterized by clusters of axons surrounded by myelin sheaths within demyelinated plaques. The staging in each lesion was performed independently by two of the authors (AN and BR).

### Immunohistochemistry

#### Apo D Detection

For immunohistochemistry, sections were treated with Triton X (0.1%, 5 min), washed in distilled water, treated with H_2_O_2_ (3%, 5 min), washed in distilled water, and treated with PBS (2 min). Non-specific binding was blocked by incubation with 1% BSA (30 min). Incubation with a specific rabbit antibody against human Apo D [1:2000 dilution, provided by Dr. Carlos López-Otín, Universidad de Oviedo (López-Boado et al., 1994; Navarro et al., 1998, 2004; Ordoñez et al., 2006)], was carried out overnight at 4°C. After several washes in PBS, sections were incubated at room temperature using a biotinylated horse universal antibody (Vector, PK-8800) (1:40 dilution, 30 min). Afterward, sections were treated with extravidin labeled with HRP (Sigma Extra-3, Sigma Chemical Co). The peroxidase activity was visualized with 0.05% diaminobenzidine (DAB) (D5637, Sigma Chemical Co) in 50 mM Tris buffer pH 7.6, containing 0.04% H_2_O_2_ (33%). Sections were counterstained with a modification of formaldehyde thionin method (Reynolds et al., 1994; Tolivia et al., 1994; Vazgiouraki et al., 2016), dehydrated, cleared in eucalyptol, and mounted with Eukitt. For controls, representative sections were processed in the same way with 1% BSA in place of the primary antibody. In addition, control sections were also incubated with Apo D antibody preabsorbed with Apo D immunizing peptide (80 μg/ml). Under these conditions, no specific immunostaining was observed.

Before quantification, we took advantage of the fluorescent qualities that myelin displays in formalin fixation to choose different areas in demyelination plaques: active, inactive, and remyelinization areas. For the quantification of Apo D presence, the chromogenic signal was selected with Adobe Photoshop CS 8.0.1 (Adobe Systems Inc., CA, United States) and quantified with ImageJ 1.37c (National Institutes of Health, Bethesda, MD, United States) software according to a procedure developed by our group (Tolivia et al., 2006). Six random regions per case (**Table 1**) were photographed using a 20× lens.

#### Double Immunostaining for Apo D and NF-200

A double immunostaining for Apo D protein and neurofilament (NF-200) was carried out according to the following protocol. The sections were treated sequentially with Triton X-100 (0.1%, 5 min), washed in distilled water, treated with H_2_O_2_ (3%, 5 min), washed in distilled water again, and treated with PBS for 2 min. Non-specific binding was blocked by incubation with 1% BSA (30 min). Incubation with a specific rabbit antibody against Apo D (1:2000 dilution) was carried out overnight at 4°C. The immunoreactivity was detected with an Extravidin–biotin–alkaline phosphatase staining kit (Sigma Extra-1A, Sigma Chemical Co). Enzymatic activity was shown by incubation with Vector blue substrate (Vector SK-5300; Burlingame, CA, United States). Slides were rinsed in PBS, placed in a plastic Coplin jar filled with 0.01 M sodium citrate buffer (pH 6), and incubated in a household microwave oven. Microwave treatment involves completely blocking contaminating staining in the double-labeling technique using primary antibodies from the same species and the same secondary antibody [see Lan et al. (1995)]. Then, incubation with a specific monoclonal antibody against NF-200 (Novocastra, NCL-NF200, 1:100 dilution) was carried out overnight at 4°C. The immunoreactivity was detected using the Extravidin–biotin–peroxidase Staining kit (Sigma Extra-3, Sigma Chemical Co). After several washes, peroxidase was visualized with 0.05% DAB (D5637, Sigma Chemical Co) in 50 mM Tris buffer (pH 7.6), containing 0.04% H_2_O_2_ (33%). Finally, sections were mounted in aqueous mounting medium. For controls, representative sections were processed in the same way with 1% BSA in place of the primary antibody. In addition, control sections were also incubated with Apo D antibody preabsorbed with Apo D immunizing peptide (80 μg/ml). Under these conditions, no specific immunostaining was observed.

#### Double Immunostaining for Crystallin and Apo D

The double immunostaining for crystallin and Apo D protein was carried out according to the following protocol. The sections were treated sequentially with Triton X-100 (0.1%, 5 min), washed in distilled water, treated with H_2_O_2_ (3%, 5 min), washed in distilled water again, and treated with PBS for 2 min. Slides were rinsed in PBS, placed in a plastic Coplin jar filled with 0.01 M sodium citrate buffer (pH 6), and incubated in a household microwave oven. Then sections were washed in PBS for 2 min and non-specific binding was blocked by incubation with 1% BSA (30 min). Incubation with a specific monoclonal antibody against alpha B crystallin (Novocastra, NCL-ABCrys-512) (1:200 dilution) was carried out overnight at 4°C. Sections were incubated with a biotinylated horse universal antibody (Vector, PK-8800) (1:50 dilution, 30 min). Afterward, sections were incubated with extravidin (Sigma Extra-3, Sigma Chemical Co), and peroxidase activity was visualized by incubation with Sigma Fast DAB (Sigma D4 168, Sigma Chemical Co) at room temperature for 30 min. After that, slides were rinsed in PBS, placed into a plastic coplin jar filled with 0.01 M sodium citrate buffer (pH 6), and heated twice for 5 min at 700 W in a household microwave. Microwave treatment involves completely blocking contaminating staining in the double-labeling technique using primary antibodies from the same species and the same secondary antibody [see Lan et al. (1995)]. Immunostaining for Apo D was carried out as previously described and, after several washes in PBS, sections were incubated for 30 min at room temperature in biotinylated horse universal antibody (Vector, PK-8800) diluted 1:50. Sections were subsequently incubated with streptavidin labeled with Fluorolink Cy3 (Amersham, PA-43001). The sections were not counterstained and were mounted in aqueous mounting medium. For controls, representative sections were processed in the same way with 1% BSA in place of the primary antibody. In addition, control sections were also incubated with Apo D antibody preabsorbed with Apo D immunizing peptide (80 μg/ml). Under these conditions, no specific immunostaining was observed.

Final images were obtained by the digital superposition of the corresponding DAB (crystallin signal) and fluorescence (Apo D signal) images of the same sections. The positive signal of each image was selected according to the method of Tolivia et al. (2006); crystallin DAB signal was converted to green and Apo D signal to red channel of a new RGB picture. This new image shows the Apo D fluorescence signal in red and the crystallin fluorescence signal in green.

Finally, a double immunohistochemical technique for crystallin and the specific astrocytic marker, GFAP, was performed in order to study the possible crystallin expression in other CNS cells. The immunohistochemistry was carried out according to the method described above; a specific monoclonal antibody against alpha B crystallin visualized by Sigma Fast DAB and a specific polyclonal antibody against GFAP (Sigma; G-9269; 1:500 dilution, Sigma Chemical Co) visualized by streptavidin labeled with Fluorolink Cy2 (Amersham, PA-42001) were used. Final images were obtained by the digital superposition of the corresponding DAB (crystallin signal) and fluorescence (GFAP signal) images of the same sections. Crystallin DAB signal was converted to red and GFAP signal to green channel of a new RGB picture. This new image shows the GFAP fluorescence signal in green and the crystallin fluorescence signal in red.

### Microscopy Observation

Sections were observed using an Epi-Fl Nikon Eclipse E400 microscope equipped with Plan-Fluor objectives, and images were recorded by a digital camera (NikonDN-100).

### Statistical Analysis

The data in the graphs are presented as the mean ± SEM. All statistical calculations were conducted using SPSS 21.0 for Windows. The test of Kolmogorov–Smirnov with the correction of Lilliefors was used to evaluate the fit of the data to a normal distribution and the test of Levene to evaluate the homogeneity of variance. Significance was analyzed by one-way ANOVA test followed by a Tukey’s test for multiple comparisons, or a Student’s *t*-test. Significant differences were considered when *p* < 0.05.

## Results

### Identification and Characterization of Focal Demyelinating Plaques

**FIGURE 1:**
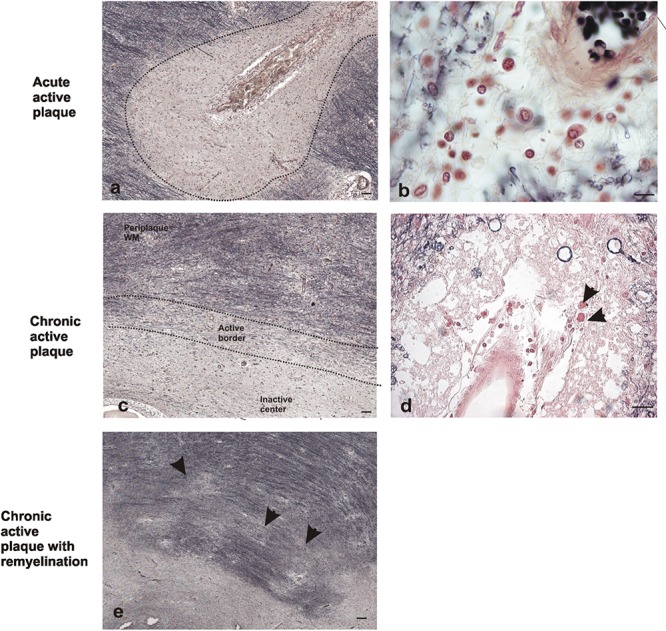
Representative images of histochemical characterization of lesions with ferric hematoxylin. Acute active plaque arround a vessel where no completely loss of myelin was found in a male patient of 58 years old **(a)**. Extravasated mononucleated cells in acute active plaque of the same patient. Scale bar: 10 μm (100×) **(b)**. Chronic active plaque with three characteristic areas in a female patient of 48 years old: periplaque WM, border area, and inactive center plaque. Scale bar 50 μm (4×) **(c)**. Macrophages around vessels in chronic active plaque edge (arrows). Scale bar: 50 μm (20×) **(d)**. Chronic active plaque with remyelination in a male patient of 58 years old, shadow plaques are also observed (arrowheads). Scale bar 50 μm (4×) **(e)**.

In order to analyze the Apo D expression in MS lesions, first of all we localized and characterized demyelination plaques in myelin stained sections according with previous studies (Lucchinetti et al., 1999; Bramow et al., 2010; Popescu et al., 2013). Different plaque types and stages of demyelinating activity were found. Acute active plaques were observed as partially demyelinated lesions with abundant and widely distributed macrophages containing myelin debris, and reactive astrocytes. Also many lymphocytes, as well as, other immune cells appeared in these lesions (**Figures 1a,b**). Chronic-active plaques were more frequently detected than acute active ones probably due to the high number of patients with progressive MS. Chronic-active plaques consist in big plaques with an inactive center and one or more expansive edges with macrophages inside (**Figures 1c,d**). In some cases, chronic-active plaques with remyelination can be observed (**Figure 1e**).

The staining for myelin not only allowed us to delimit the chronic-active plaques but also to distinguish different morphological areas within lesions: (Ramagopalan et al., 2010) periplaque WM area, the surrounding WM that seems not to be affected by demyelination (McQualter and Bernard, 2007) plaque active area, containing myelin-laden macrophages and partially demyelinated (Zindler and Zipp, 2010), and plaque inactive center with a drastic reduction or even absence of myelin (**Figure 1c**). Since 27% of our cases showed remyelination features, a new region called remyelinating area (McGuire et al., 2011) was incorporated to the study. This area is characterized by the existence of small packages of newly formed myelinated nerve fibers crossing the demyelinated region.

### Apo D Expression Pattern in MS Plaques

**FIGURE 2:**
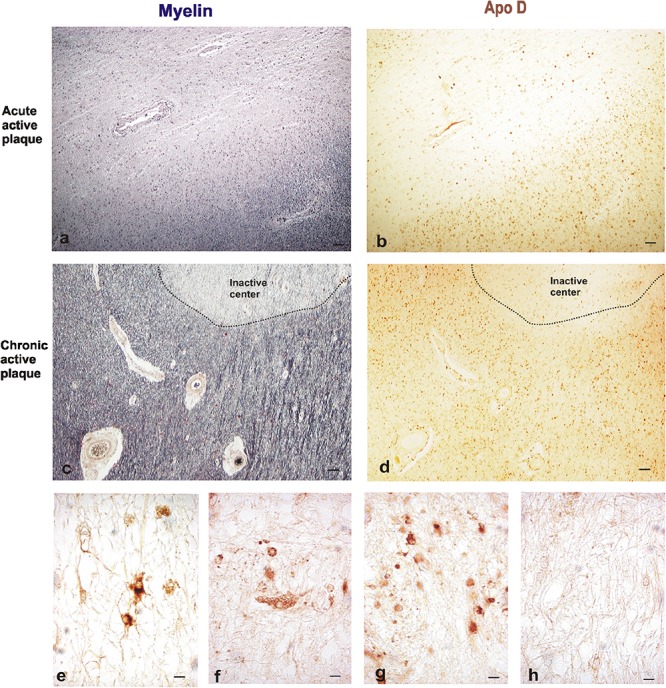
Representative images of histochemical and immunohistochemical comparison of different lesion areas in a chronic active plaque. Focal active demyelinated lesion in periventricular WM **(a,b)**. Consecutive sections of a male patient, 35 years old. **(a)** Myelin stain with ferric hematoxylin. **(b)** Immunohistochemistry for Apo D. Expanding rim of a chronic active plaque surrounding an inactive area **(c,d)**. Consecutive sections of a male patient, 68 years old. **(c)** Myelin stain ferric hematoxylin. **(d)** Immunohistochemistry for Apo D. Scale bar 50 μm (4×). Immunopositive cell types in plaques. Reactive astrocytes with large cytoplasm, round nuclei, and stellate neurites **(e)**. Macrophagic cell with numerous phagosomes inside **(f)**. Many small OLGs with small round nuclei, scarce cytoplasm, and one or two projections **(g)**. Negative immunostaining in inactive plaque **(h)**. Scale bar 10 μm (100×).

In order to analyze the Apo D implication in demyelination and remyelination processes in MS, we analyzed the Apo D expression pattern in focal demyelinating lesions by immunohistochemistry techniques (**Figures 2a–d**).

The active plaque areas show a low overall Apo D signal when we compare with the intact periplaque WM. However, reactive astrocytes and active macrophages of these areas appear intensely labeled for Apo D (**Figures 2e,f**); most astrocytes positives for this protein exhibit round-shaped cell bodies, hypertrophy, and numerous fibrillary processes (reactive astrocytes).

In the case of chronic plaques, Apo D expression was again higher in periplaque WM than in the inactive plaque area (**Figures 2g,h**). This fact may be due to the large amount of Apo D-positive cells present in these surrounding areas as well as the intensity of their labeling (**Figure 2g**).

**FIGURE 3:**
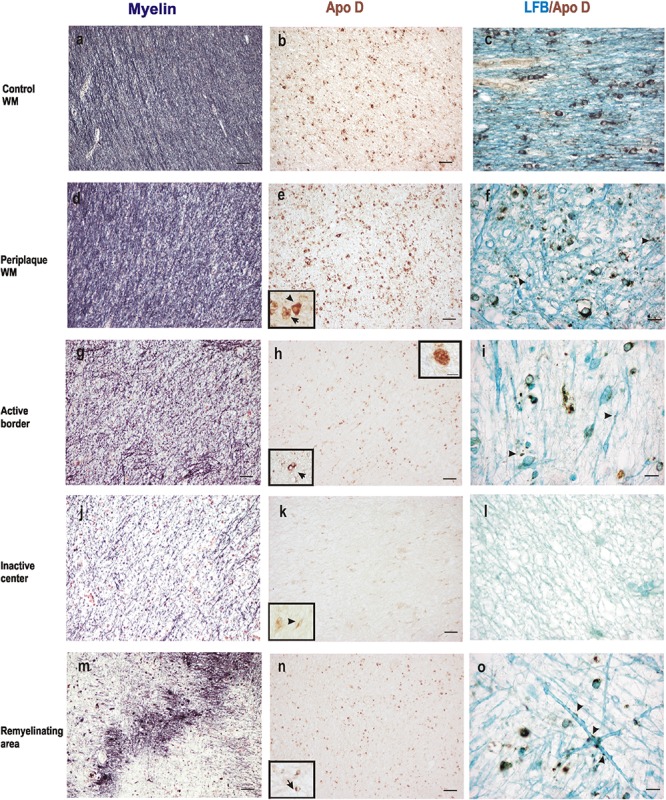
Representative images of histochemical and immunohistochemical comparison of different lesion areas in a chronic active plaque. Control WM of male control subject of 66 years old. **(a)** Characteristic myelin pattern in WM area. **(b)** Consecutive section immunostained for Apo D. **(c)** Colocalization of myelin and Apo D immunohistochemistry in cytoplasm and processes of OLGs. Female subject of 52 years old with MS **(d–o)**. Periplaque WM. **(d)** Characteristic myelin pattern in periplaque area. **(e)** Consecutive section immunostained for Apo D showing mainly positive OLGs (arrow), but also astrocytes (arrowhead) as it is shown in the insert. **(f)** Colocalization of myelin and Apo D immunohistochemistry in cytoplasm and processes of OLGs. Apo D containing granules were found surrounding myelin sheath. Active area. **(g)** Active demyelination can be observed in this area. **(h)** Intensity of the immunostaining for Apo D is weaker than in the periplaque WM. Apo D positive macrophagic cells (top insert) and less OLGs can be observed in this area (bottom insert). **(i)** Double stain for myelin and Apo D. The arrowhead points to the granular content of distal processes of OLGs around myelin sheath. Inactive area. **(j)** Myelin stain shows a completely demyelinated area. **(k)** This area is negative for Apo D with the exception of a few astrocytes with a weak staining (insert). **(l)** No myelin nor Apo D label can be found in inactive plaque area. Remyelination area in a sclerosis plaque. **(m)** A package of myelinated axons passing by a demyelinated area. **(n)** Consecutive section immunostained for Apo D. Positive OLGs can be observed in this area (arrow in the insert). **(o)** Myelinated axons stained with LFB also present granular Apo D immunostaining surrounding (arrowheads). Scale bars: **(a,b,d,e,g,h,j,k,m,n)**, 30 μm (10×); **(c,f,i,l,o)**, 10 μm; (Inserts), 10 μm (100×).

When we analyze myelin of chronic MS lesions with a specific stain for this protein we found that, as it was to be expected, myelin was reduced to low levels in active areas and to very low levels in inactive ones, compared to control WM area and periplaque WM (**Figures 3a,d,g,j**). Interestingly, the Apo D immunosignal showed a similar pattern than that of myelin. Thus, the immunoreactivity for Apo D tended to decrease as we approached the plaque border and even more in the demyelinated plaque area (**Figures 3b,e,h,k**). Based in the cell morphology, we observed that OLGs, macrophagic cells, and some isolated astrocytes (inserts in **Figures 3e,h**) were responsible for the expression of Apo D in all these areas (**Figures 3b,e,h,k,n**). In the remyelinating regions, both myelin and Apo D levels increase in line with the remyelination processes that are taking place (**Figures 3m,n**).

Finally, co-localization studies of myelin and Apo D proteins were performed in immunohistochemistry tissue sections counterstained with LFB (**Figures 3c,f,i,l,o**). We found that Apo D appeared preferentially in the cytoplasm and distal cell processes of OLGs in all the plaque types and patients studied. Small granules containing Apo D could also be observed around the myelin sheath of these cells (**Figures 3c,f,i,o**). Noteworthy, in the border of plaques, even with low levels of myelin, some remaining OLGs showed immunolabeling for Apo D (**Figure 3i**). OLGs expressing Apo D reappeared in focal remyelination areas, specifically in those where new myelin envelopes are being formed (**Figure 3o**).

### Quantification of Apo D Levels in Chronic MS Plaques

**FIGURE 4:**
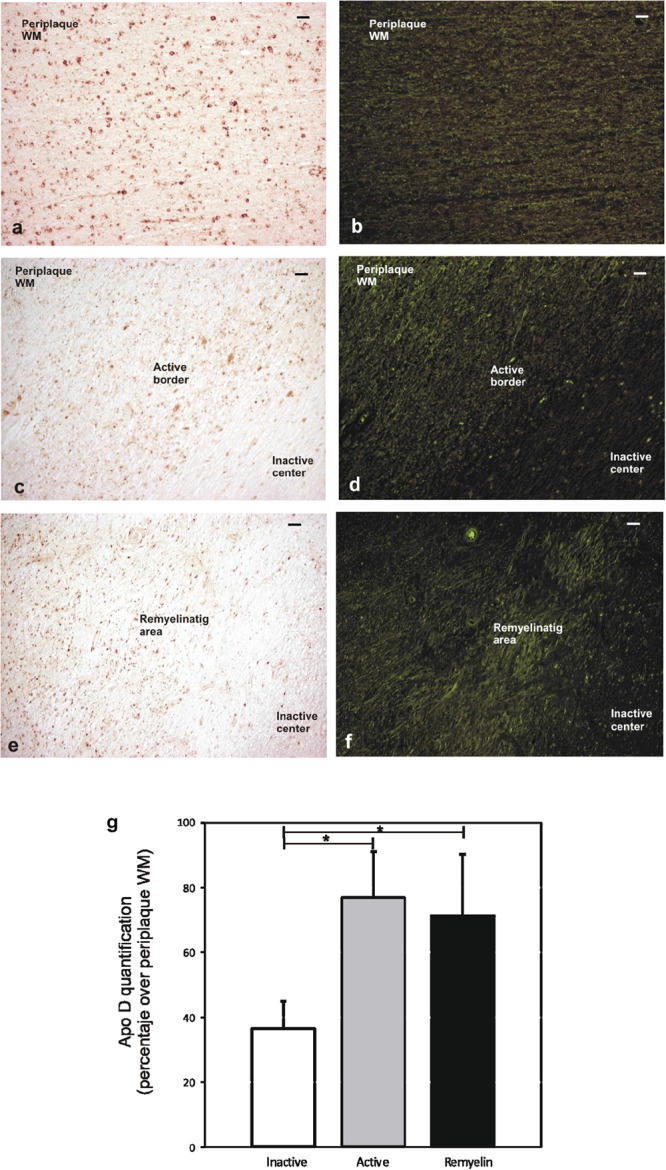
Representative image of remyelination area in a sclerosis plaque. Immunohistochemistry for Apo D visualized under light microscopy **(a,c,e)**, and same area observed with fluorescent microscopy to visualize autofluorescence of myelin sheaths **(b,d,f)**. Scale bar: 50 μm (10×). Histogram showing densitometric quantification of Apo D **(g)**. Bars represent mean density in 20× field ± SEM. ^∗^Statistically significant differences, *p* < 0.05. Periplaque WM was taken as 100% of Apo D and the rest of the values relativized to it.

The quantification of Apo D protein level in the most frequent and better defined chronic plaques showed clear discrepancies between the different areas under study. Since it was difficult to distinguish plaque areas in immunostaining sections for Apo D, we used the intrinsic fluorescent properties of myelin to unequivocally detect them and improve the quantification process (**Figures 4a–f**). The data showed statistically significant differences in the Apo D burden between inactive (36.5 ± 8.2) and active areas (76.6 ± 14.3) in chronic plaques (*p* < 0.05), and also between inactive and remyelination areas where Apo D recovers (71.25 ± 18.7; *p* < 0.05), taking periplaque WM as 100% expression (**Figure 4g**). However, we did not find significant differences when we compared active with remyelination areas (**Figure 4g**).

### Apo D and Crystallin Co-localization

Our results clearly demonstrated a decrease in the Apo D content from the active area to the chronic inactive one in parallel with the loss of myelin in all the plaque types and patients studied. However, an important issue still to be determined is the exact relationship between Apo D levels and the number of OLGs present in these areas. For this purpose, first we tested different OLG markers and we found that crystallin (Pountney et al., 2005; Patel and Balabanov, 2012; Mallucci et al., 2015) was the best since it was able to unequivocally detect OLGs in tissues without myelin interactions, corroborating our glial cell identification based on their specific morphological characteristics (data not shown). Second and in order to confirm that crystallin is expressed specifically in OLGs, a double immunohistochemical technique for crystallin and the specific astrocytic marker GFAP was performed. Our results clearly demonstrated no colocalization between these two markers and, consequently, that crystallin immunoreactive cells are in virtually all case OLGs (**Supplementary Figure S1**). In fact, only a very few astrocytes were positives for crystallin so these cells have not been taken into account in our studies.

**FIGURE 5:**
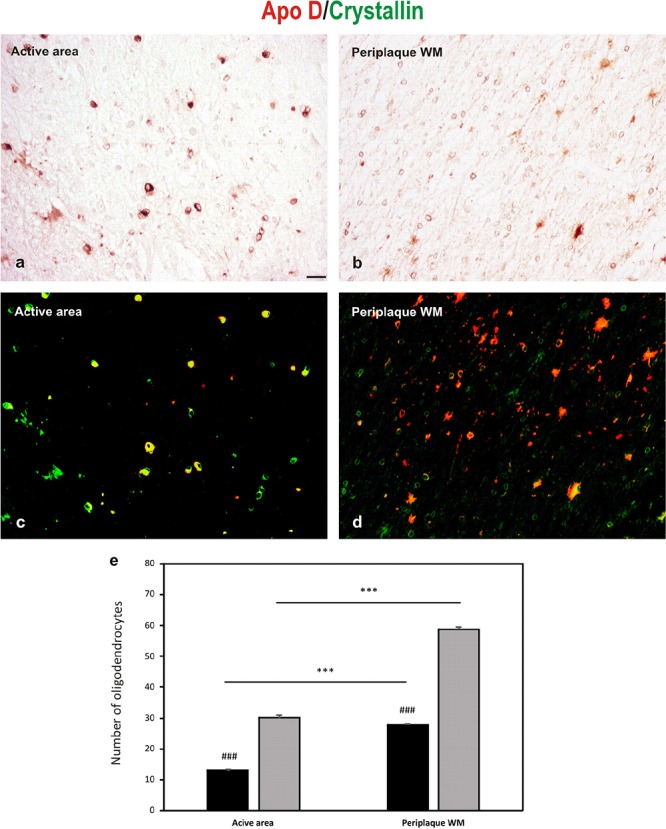
Double immunohistochemical technique for crystallin (DAB signal, brown) and Apo D (Cy3 fluorescence signal, red). **(a,b)** The bright-field micrographs of crystallin signal **(c,d)** The digital superposition, on those section, of fluorescence signal for Apo D. Digital superposition shows clearly the absence of Apo D expression in some OLGs (green signal) and OLGs with Apo D expression (yellow signal). Representative image of active plaque area **(a,c)**. Representative image of periplaque WM area **(b,d)**. Scale bar: 20 μm. Histogram showing number of OLGs positive for crystallin (gray bars) and also labeled for Apo D (black bars; colocalization signal) **(e)**. Bars represent OLGs number in 20× field ± SEM. ^∗∗∗^Statistically significant differences active area *versus* periplaque WM, *p* < 0.001. ^###^Statistically significant differences Apo D *versus* crystallin, *p* < 0.001.

Double immunostaining for Apo D and crystallin is showed in **Figure 5**, in both representative images (**Figures 5a–d**), and the quantification of OLGs negative for Apo D (only positive for crystallin) and positive for Apo D (colocalization between crystallin and Apo D signals) (**Figure 5e**). A statistical significant reduction in the number of OLGs expressing Apo D (colocalization signal) in the active area (13.04 ± 0.43) compared with the periplaque WM (27.8 ± 0.45; *p* < 0.001), and a significant number of OLGs do not express Apo D in the periplaque WM (31 ± 0.50 vs. 58.75 ± 0.94; *p* < 0.001) as well as in active areas (17.1 ± 0.6 vs. 30.1 ± 1; *p* < 0.001) was found. In fact, we have found that not all the OLGs are Apo D positives, which becomes more evident in active demyelinating areas in which there also seems to be more colocalization (**Figures 5a–d**).

### Apo D Expression and Axonal Preservation

**FIGURE 6:**
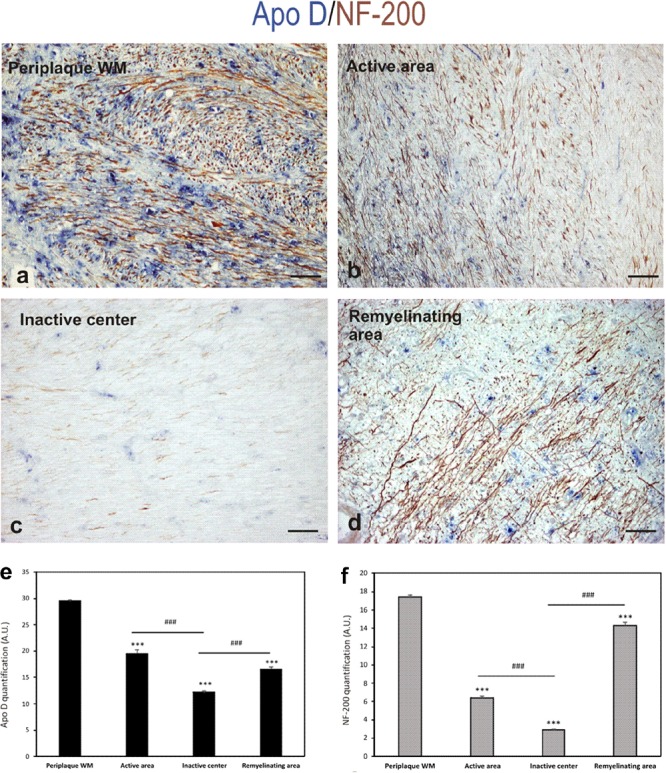
Double immunohistochemical technique for Apo D (blue) and NF-200 (brown). The representative micrographs show a chronic plaque where it can be clearly distinguish the surveyed areas: periplaque WM area **(a)**, active area **(b)**, inactive area **(c)**, and remyelination area **(d)**. Scale bar: 60 μm. Histogram showing densitometric quantification of Apo D **(e)**. Histogram showing densitometric quantification of NF-200 **(f)**. Bars represent mean density in 20× field ± SEM. ^∗∗∗^Statistically significant differences *versus* periplaque WM, *p* < 0.001. ^###^Statistically significant differences inactive center *versus* active area, *p* < 0.001. AU: arbitrary units.

In the light of the described results and the neuroprotective role suggested for Apo D, we used a double immunostaining for Apo D and NF-200 to study whether the apolipoprotein expression could be related with axonal preservation. As we expected, the expression pattern for both proteins was similar. In fact, the immunolabeling for Apo D and NF-200 statistically significant decreases in the chronic inactive plaque areas (12.18 ± 0.3 and 2.9 ± 0.07, respectively) compared to periplaque WM (29.5 ± 0.4 and 17.4 ± 0.23, respectively; *p* < 0.001) (**Figures 6c–f**). Notably, we have observed that the amount of Apo D and neuronal NF-200 decreased, in a statistical significant way, in parallel from the edge (19.55 ± 0.7 and 6.4 ± 0.22, respectively) to the inactive plaque center (12.18 ± 0.3 and 2.9 ± 0.07, respectively; *p* < 0.001) (**Figures 6a–c,e,f**). Of note is that many axons conserved immunostaining of NF-200 in active demyelination areas even in absence of Apo D expression (**Figure 6B**). In remyelination areas, both signals were again found (16.58 ± 0.5 and 14.32 ± 0.40, respectively) (**Figures 6d–f**).

## Discussion

While much progress has been achieved on the involvement of Apo D in some neurodegenerative diseases in the past years, little is known about its role in MS. The neuroprotective and antioxidant effects postulated for this apolipoprotein and its proved overexpression in glial cells during demyelinating/remyelinating processes are fundamental reasons to suspect that Apo D could be implicated in MS. The immunohistochemical characterization of Apo D expression pattern in the anatomopathological classical features of MS, sclerosis plaques, is a first step necessary to address this goal.

In this work, we have observed that Apo D expression is further reduced in MS lesions respect to the unaffected WM, in all the plaque types and patients studied. In this sense, an interesting finding is that there are labeling differences within the plaque, significantly lower Apo D expression in the inactive area than in the active one which increases again in remyelination areas. Remarkably, the immunohistochemical approaches reveal a possible relationship between OLGs, myelin, and Apo D expression in MS plaques. As it was expected, myelin protein levels decrease in demyelination plaques from the periplaque WM to the border of the lesions an even more to the center. In fact, this loss of myelin sheets wrapping myelinated nerve fibers is closely related with OLGs degeneration that characterize MS pathophysiology (Zindler and Zipp, 2010; Popescu et al., 2013). The Apo D expression pattern in both active and chronic plaques was similar than the one found for myelin. Apart from myelination, mature OLGs as wells as OPCs are the cells primarily responsible for the Apo D synthesis in the normal WM of adult human brain, during CNS embryogenesis and in myelin sheaths formation (Hu et al., 2001; Navarro et al., 2004). Bearing in mind these data, one could think that the Apo D downregulation observed in sclerosis plaques is consistent with OLGs death caused by an exacerbated autoimmune reaction. However, we have demonstrated that not all OLGs express Apo D in periplaque intact regions, which becomes more evident in demyelinating areas. The OLG degeneration only could explain, in our opinion, one part of the reduced Apo D levels, what pathological processes of MS compromise, directly or indirectly, the expression of Apo D by the intact OLGs could explain the rest (Patel and Balabanov, 2012).

Regarding the role of Apo D in myelination/remyelination processes, literature indicates that Apo D is a secreted lipocalin that takes place in a neuroprotective mode in various pathophysiological mechanisms of CNS and PNS, almost always in relation with its ability to selectively bind different small lipid molecules (Rickhag et al., 2008; Kosacka et al., 2009; García-Mateo et al., 2014). In this sense, Apo D is significantly up-regulated in Schwann cells and macrophages of sciatic nerve after crush injury (Boyles et al., 1990; Spreyer et al., 1990) where it may promote recovery locomotor function after damage, myelin clearance, and regulate the number of macrophages recruited to the injury site, which means controlling the magnitude and timing of the inflammatory response (Ganfornina et al., 2010; García-Mateo et al., 2014). In fact, the lack of Apo D seems to compromise myelin compaction but more importantly myelin clearance after injury, resulting in a delayed axonal regeneration/remyelination (Ganfornina et al., 2010; García-Mateo et al., 2018). In addition, Apo D gene is among the regeneration-associated genes whose expression is up-regulated in rat dorsal root ganglia following chronic constriction injury of the sciatic nerve (Kim et al., 2001). Despite the function of Apo D in myelin homeostasis in the PNS is well known, its exact position during myelination/demyelination processes of the CNS remains unclear.

Apo D is expressed in the development of the CNS and increases during aging and in some neurodegenerative diseases (Navarro et al., 2004; Muffat and Walker, 2010; Sanchez et al., 2015). Gene profiling analysis of ischemic and stroke rat brain tissues showed the up-regulation of genes related to lipid transport, including Apo D, mainly in peri-infarct and WM areas in cells identified as mature OLGs and reactive astrocytes (Rickhag et al., 2008). It has been proposed that Apo D may exert a neuroprotective influence in the acute stroke phase and may also support neuronal regeneration and remyelination in the extended post-stroke recovery phase (Rickhag et al., 2008). Demyelination, neuronal growth, and remyelination are among the multiple events that take place in MS brains and that require a synthesis and recruitment of lipids to form plasma membranes and myelin sheaths. Noteworthy, we have found that the expression of Apo D, one of the most important glial-derived proteins in the myelin dynamics, decreases in the sclerosis plaques of MS brains. On one hand, we have observed Apo D label in cytoplasm and distal processes of OLGs but no colocalization with myelin itself. On the other hand, the decrease in Apo D protein is not only due to the lower number of OLGs inside the plaques but many of these OLGs are not able to express it. Activation of the inflammatory cascade in MS ultimately compromise, directly or indirectly, OLGs function and viability (Patel and Balabanov, 2012). In this sense, it has been shown that inflammatory mediators such as cytokines, that is, IL-1, released from microglia/macrophages and astrocytes could alter Apo D expression in mature OLGs (Rassart et al., 2000; Clausen et al., 2005). Probably this exacerbated environment in the early stages of MS leads to defects in modulating the Apo D expression in astrocytes and OLGs which, together with the death of these latter, may affect the demyelination/remyelination balance turning it less effective, and contributing to the development of the disease.

Also of interest is highlighting that Apo D disappears in demyelinated plaques but returns when remyelination occurs. According with our findings, a correlation between Apo D and loss of axonal processes in the sclerosis plaques could be ruled out. Although more studies are needed in this respect, one plausible explanation may place Apo D in the molecular and cellular mechanism that control spontaneous remyelination after damage. As mentioned above, Apo D is expressed mainly by mature OLGs as wells as OPCs in the brain which, in turn, are ultimately responsible for the restoration of new myelin sheaths in demyelinated axons in MS (Hu et al., 2001; Bradl and Lassmann, 2010; Clemente et al., 2013; de Castro et al., 2013). Indeed, it seems that adult OPCs react to damage and are able to proliferate, migrate, and differentiate in response to mitogens and pro-migratory factors secreted by astrocytes and microglial cells to replace lost OLGs (Clemente et al., 2013). Some of the neuroprotective functions of Apo D in this context could be related with recent findings that suggest that it may regulate the migratory behavior of different motile cells in association to growth factors (Dentelli et al., 1999; Leung et al., 2004; Pajaniappan et al., 2011; Elo et al., 2012). A possible link between Apo D expression/recapture and OPCs differentiation is another factor to take into account. The Apo D implication in cell differentiation has been assessed previously in both *in vivo* and *in vitro* studies (López-Boado et al., 1994; Kosacka et al., 2009). The data showed that Apo D may act as neurotrophic factor that promotes neurite outgrowth and synaptogenesis in dorsal root ganglion neurons (Kosacka et al., 2009). Interestingly, it has been also shown that Apo D seems to mediate neuronal differentiation in a retinoic acid (RA)-dependent manner in various cell lines; Apo D may act as RA carrier (Ruiz et al., 2013). Unfortunately, remyelination finally fails in this pathology most likely due to the shortage of OLGs and/or by an inadequate supportive environment for their differentiation.

Apart from OLGs and macrophages, astrocytes also express Apo D in WM. Our group and others have previously reported that in some human neurodegenerative diseases, reactive glial progression seems to be accompanied by an increase in Apo D expression being this increment much higher in astrogliosis areas, probably due to neuron injury or loss (Ordoñez et al., 2006; Bajo-Grañeras et al., 2011; Dassati et al., 2014). Accordingly, we have observed that some reactive astrocytes in active plaques show an intense immunosignal for Apo D but, surprisingly, astrocytes in the edge of plaques or even within the glial scar in the chronic plaques show a much lower signal. Since one of the postulated role for Apo D is to act as a neuroprotective and antioxidant molecule, one could expect that Apo D expression would be higher in periplaque and active areas of MS plaques. This apparent contradiction could be explained again by the influence of MS local levels of cytokines in the astrocyte protein expression profiles (Rassart et al., 2000; Holley et al., 2003).

## Conclusion

In summary, the dual function of Apo D, as a tissue specific lipid carrier and an antioxidant and neuroprotective molecule, makes it a potential versatile player in MS pathology. To our knowledge, this is the first study showing a detailed characterization of Apo D expression pattern not only in the MS lesions but also in different types of cells somehow involved in the disease. Our results seem to indicate an important function of Apo D in MS and may serve as a good starting point for its study in the myelination/remyelination of CNS. However, further investigation is needed to appropriately address the exact implication of Apo D in these processes as well as its neuroprotective potential that, if confirmed, would open new avenues in the search for alternative therapeutically approaches in the fight against demyelinating diseases as MS.

## Author Contributions

AN contributed to writing the manuscript, performed and analyzed the cytoarchitectonic staining and the immunohistochemical techniques. BR performed and analyzed the cytoarchitectonic staining. EdV performed part of the staining procedures. EM-P contributed to writing the manuscript, and assisted in analyzing the results and images processing. AA supervised the project and contributed to the anatomopathological diagnosis. JT contributed by formulating the initial hypothesis, directed the experiments performed, and contributed to scientific discussions and manuscript writing.

## Conflict of Interest Statement

The authors declare that the research was conducted in the absence of any commercial or financial relationships that could be construed as a potential conflict of interest.

## Abbreviations

AD: Alzheimer’s disease
Apo D: Apolipoprotein D
Apo E: Apolipoprotein E
BBB: blood–brain barrier
BSA: bovine serum albumin
CNS: central nervous system
CSF: cerebrospinal fluid
DAB: diaminobenzidine
HDL: high-density plasma lipoproteins
HRP: horseradish peroxidase
IL-1: interleukin 1
LFB: luxol fast blue
MS: multiple sclerosis
OLGs: oligodendrocytes
OPCs: oligodendrocytes precursor cells
PAS: periodic acid–Schiff
PBS: phosphate-buffered saline
PNS: peripheral nervous system
PPMS: primary progressive MS
RA: retinoic acid
SPMS: secondary progressive MS
WM: white matter.
