# Supplementary material for: Expression Pattern of Myelin-Related Apolipoprotein D in Human Multiple Sclerosis Lesions
Source: Front Aging Neurosci. 2018 Aug 21;10:254. doi: 10.3389/fnagi.2018.00254 (PMC6110904; doi:10.3389/fnagi.2018.00254)
Supplement: Supplementary file 1 [file Image_1.pdf]

Supplementary Figure 1

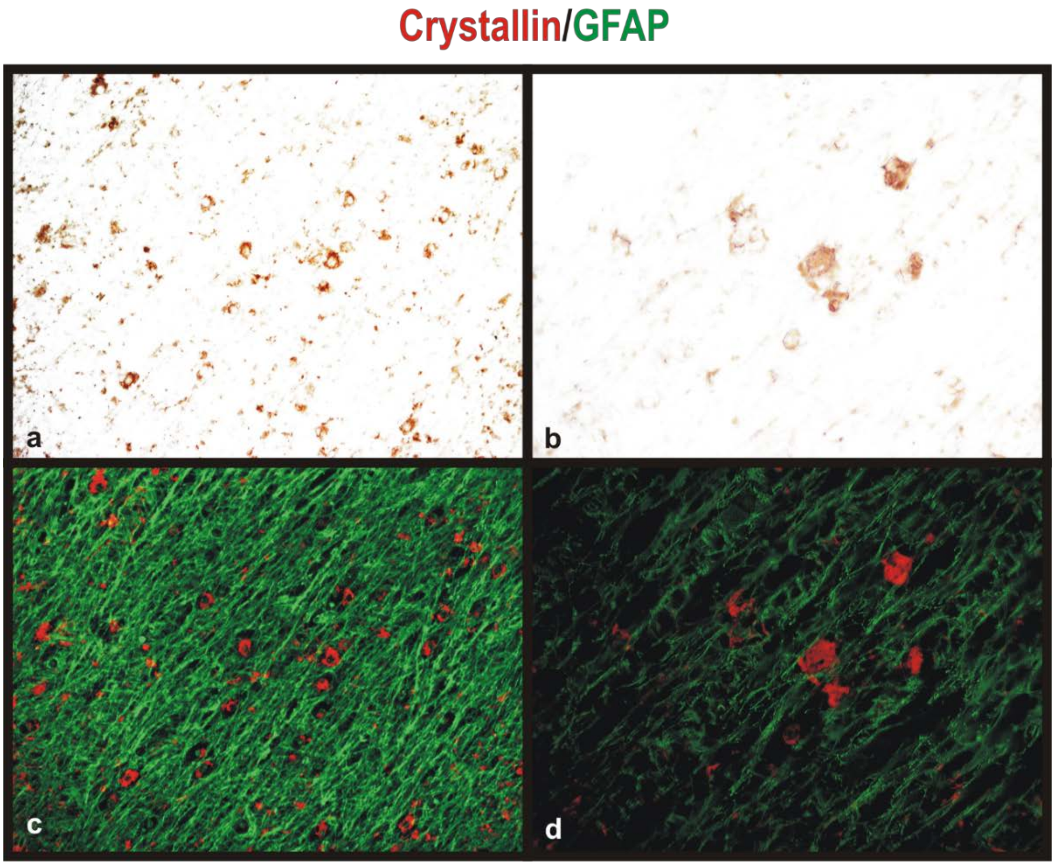

Supplementary Figure 1. Double immunohistochemical technique for crystallin (DAB signal, brown) and GFAP (Cy2 fluorescence signal, green). Representative images a, b show the bright-field micrographs of crystallin signal and images c, d show the digitally superposition, on those section, of fluorescence signal for GFAP in green and for crystallin in red. Images a, c: 40x. Images b, d: 100x.
